# Analysis of Thermomechanical Properties of Polyethylene with Cement Addition

**DOI:** 10.3390/ma15041587

**Published:** 2022-02-20

**Authors:** Adam Gnatowski, Agnieszka Kijo-Kleczkowska, Łukasz Suchecki, Paweł Palutkiewicz, Jarosław Krzywański

**Affiliations:** 1 Faculty of Mechanical Engineering and Computer Science, Czestochowa University of Technology, 42-200 Czestochowa, Poland; a.kijo-kleczkowska@pcz.pl (A.K.-K.); lukasz.suchecki@pcz.pl (Ł.S.); pawel.palutkiewicz@pcz.pl (P.P.); 2 Faculty of Science and Technology, Jan Dlugosz University in Czestochowa, 42-200 Czestochowa, Poland; j.krzywanski@ujd.edu.pl

**Keywords:** cement, polymer composites, DSC testing, microstructure, combustion, DMTA testing, tensile strength

## Abstract

The paper undertakes preliminary research towards the identification of the use of plastic waste, taking into account the possibility of increasing their mechanical strength and reducing flammability, as well as reducing the emission of harmful compounds to the atmosphere through the addition of cement. This is extremely important not only from the point of view of the wide use of plastic products in the industry, but also their thermal utilization. The present study deals with the aspect of the utilization of waste polyethylene (HDPE) as a matrix in composites with filler in the form of cement at 5 and 10%. The composite samples were prepared by injection molding after the prior proper mixing of the components. Comparative thermomechanical (DSC, tensile strength, DMTA), microstructure and flammability results are presented for HDPE samples and their composites with cement. It was found that the addition of cement as a filler to polyethylene made it possible to obtain composites with good thermomechanical properties.

## 1. Introduction

The properties and structure of polymers depend on the conditions of their use, and the addition of fillers modifies the structure of polymeric materials. The development of civilization, on the one hand, requires continuous improvement of industrial technologies for the production of various types of polymer products, but on the other hand, it is associated with an increasing volume of multiple types of waste. Most plastic waste is still landfilled, and only a small amount is recycled or thermally disposed of. Due to the growing problem with this type of waste material all over the world, a way is currently being sought for their effective and possibly the most ecological way of recycling and disposal. That is why it is essential to conduct experimental research to determine the possibilities of rational management of the said waste. The literature has addressed the use of plastic waste, such as in the production of construction materials, conversion into fuel, use in the re-manufacturing of household goods and clothing, and use as a matrix in polymer composites.

Composites are a combination of at least two components (phases) with different properties, formed using different technologies, and the obtained new materials express new properties. In polymer composites, plastics are the matrix, while the other components are the filler. For example, polymers used as a matrix in ceramic-polymer composites have the following functions: allow loads to be transferred to the fibers, give the product the desired shape, determine the thermal and chemical properties and the flammability of composites, have a significant influence on the composite manufacturing methods [1,2].

Manufacturing composites with plastics not only reduces the amount of polymer waste, which is extremely important in terms of environmental protection, but also reduces the need to consume mined fossil resources.

In the literature, researchers undertake studies on the possibility of using various types of waste to produce construction materials. These include demolition materials [3], ceramic hollow bricks [4], sanitary ceramics [5], glass fibers and waste [6], rubber waste [7], cork [8], ashes from biomass co-firing [9], and polymeric materials in concretes and cement mortars [10,11].

Noteworthy are polymer-cement composites (PCC), which are obtained by adding polymer to the concrete mixture in an amount not exceeding 5% by the weight of cement. Smaller amounts of cement are classified as admixtures [12]. Due to chemical reactivity, we distinguish post-mix polymer modifiers, i.e., chemically cured synthetic (epoxy) resins that crosslink simultaneously with cement hydration and pre-mix polymer modifiers, i.e., essentially chemically unreactive polymers (e.g., polyacrylic esters) who acts mainly physiochemically in coalescence form of polymer particles to generating a continuous polymer film. The polymer may be introduced into the concrete mixture in various forms, such as an aqueous dispersion, emulsion, re-dispersible powder (after mixing with water, it again forms a dispersion), aqueous solution, or liquid synthetic resin (binary system resin-hardener) [13]. The addition of polymer positively influences the properties of cement composite. It particularly improves flexural and tensile strength, tightness against liquids and gases (thus material durability), and adhesion to various substrates, including concrete base. These are advantages for polymer-cement concretes and mortars widely used to repair and protect concrete structures, making pavements, e.g., industrial floors and precast elements [14]. Mineral additives with pozzolanic properties, especially fly ash, are now widely used as concrete additives and as the main ingredients of common types of cement [15]. The polymer added to cement causes changes, both in the technological and functional properties of the composite [12]. Most of these properties are improved (tensile and flexural strength, adhesion to the substrate, liquid- and gas-tightness). The beneficial properties of materials with polymer-cement binders ensure that their use is constantly increasing. The main areas of application of this type of material are repair and protection of structures from corrosion, road and bridge surfaces, industrial floors, and prefabricated elements. In paper [16], the authors also presented the results of research on agrocement, showing its good strength and durability.

Paper [17] extensively deals with the types of plastics and methods used to obtain plastic aggregate, methods of evaluating different properties of aggregate and concrete, properties of plastic aggregates, fresh and hardened concrete of cement mortar and concrete in the presence of plastic aggregate, and the use of plastic waste in concrete production.

Wang and Meyer [18] investigated the possibility of using recycled high-impact polystyrene (HIPS) as a substitute for sand in cement mortar. It was shown that the compressive and tensile strength of mortar decreases by replacing sand with HIPS, but the decrease in tensile strength is much less. HIPS makes the mortar more ductile. HIPS reduces dry density, dynamic modulus of elasticity, thermal conductivity, and water vapor permeability, but does not affect freeze-thaw resistance. The use of mortar made with different HIPS contents is promising, mainly due to better thermal insulation.

The authors of the paper [19] presented a study on PET, PP and other similar plastics used in reinforced concrete, mortars, and concrete mixes instead of natural materials such as sand. It was shown that the addition of plastics at 0.1% to 0.5% increases the durability of the mix by up to 14.28%. The tensile strength can be increased by 1.63% up to 23.6% by adding plastic to concrete at 0.5% to 1.5% by volume of the mix. However, some research results indicate that replacing 5% of the sand with plastic can cause a deterioration of up to 8% in flexural strength while replacing 15–50% causes up to an 18% decrease in these parameters. Researchers using SEM (scanning electron microscopy) methodology agree that 60% replacement of sand with plastic results in improved thermal conductivity up to 58%, while subsequent addition of even 1% plastic changes the properties of the mixture, reducing its thermal conductivity up to 18%.

According to [20], it is possible to manufacture plastic cement from polyethylene waste and Portland cement, in proportions of 60% and 40%. It was found to reduce its density, increase plasticity, and improve workability, i.e., produce lightweight materials. The density increases as the proportion of waste increases to 30% and then gradually decreases. The maximum density is 1.972 gm/cm^3^, being lower than the density of cement mortar from sand and Portland cement. The moisture content of the plastic cement produced in this study ranged from 10.5% to 23.4% for the product immersed in water for seven days. However, for products immersed for 28 days, the moisture content decreased to levels in the range of 3.6% to 11.6%. The best moisture percentage was equal to 3.6 and 3.79% for products with 25% and 30% waste polyethylene, respectively. The best compressive strength for the product was found in the mixture of 25%, 30%, and 35% polyethylene. Their yield strengths are 971, 915, and 945 N for seven days of immersion in water, and 2352 for the 25% blend and 1271 N 30% blend, respectively, after 28 days of immersion. Products containing 25% to 30% waste polyethylene have good workability, allowing for trouble-free hole drilling. Based on the research, it has been shown that the optimum percentage of waste polyethylene is between 25 and 35%, which gives good mix properties.

It is important to more carefully identify cement as an additive to plastics, mainly in terms of economy (for example low cement price compared to other flame retardants) and ecological (cement is a mineral binder).

In the articles dealing with the addition of mineral and inorganic substances to plastics [21,22,23,24,25,26,27,28,29], it was shown, inter alia, that they can act as reinforcing fillers, processing aids, impact modifiers, and they are used to increase the polymeric material’s modulus of elasticity and stiffness. In addition, flame retardants (including cement) used as additives to plastics should slow down the combustion process and smoke emission.

For example, in paper [24] the authors showed that talc enhances the composites’ thermal conductivity and thermo-physical properties. No significant effect on the composite tensile yield and fracture strength was observed. The thermal composites’ conductivity, thermal diffusivity, and specific density values increased almost linearly.

The authors of paper [25] showed that incorporation of carbon black in HDPE emphasizes the crystallinity and crystallized size more than that of inorganic filler-talc. The tensile strength of the composite decreases with the increase of both types of fillers, and this decrease is explained on the basis of the Nielson model. Flexural strength increased with the increase of carbon black content but decreased with the increase of talc content.

In the paper [26], the authors stated that, poor thermal properties are one of the main limitations in the application of polymers. Thus, HDPE/10% CaCO_3_ nanocomposite was manufactured. The properties of HDPE and its nanocomposite were examined through differential scanning calorimetery (DSC) and thermo mechanical analyzer (TMA) tests. DSC tests showed that the addition of nano-sized calcium carbonate to HDPE caused an increase in heat capacity, sensible heat, and crystallinity index. The TMA results illustrated an increase in dimensional stability of HDPE as nano-sized calcium carbonate was added to it.

Some authors [27] showed that the presence of CaCO_3_ does not have a considerable effect on the melting properties of the composites. TGA analyses showed that the thermal stability of the composites is better than the neat HDPE resin.

In paper [28], authors tested high-density polyethylene, which was filled with chalk in various concentrations ranging from 10% to 60% by weight. Ethylene oxide oligomer Mw = 300 was used as a liquid modifier for chalk in the filler amount of 0 ÷ 20 wt %. The mechanical properties of these composites showed that high-density polyethylene filled with chalk have quite high ultimate elongation and impact strength while their elastic modulus and tensile strength are very near to those values for pure high-density polyethylene. On the basis of mechanical properties and microscopical observations, the crack and microcrack damping is attributed to the presence of an ethylene oxide oligomer.

The authors of paper [29] showed that kaolin, mica, talc and calcium carbonate are the most often used as fillers to reduce the production cost and to improve the properties of the thermoplastics, including electrical and thermal conductivity hardness, strength, and rigidity, flexural modulus, dimensional stability, and crystallinity.

In this study, the effect of the cement filler, in the amounts of 5% and 10% (by weight), on the thermomechanical properties of HDPE moldings was analyzed. Thermal tests by DSC and flammability tests by UL 94 V, UL 94 HB methods, microstructure tests, as well as tensile strength tests and DMTA measurements, were performed.

## 2. Materials and Methods

To carry out the tests, post-production waste in the form of granules of plastic produced by Lotte Chemical with trade name Hivorex 2210J was used, processed in accordance with the manufacturer’s data (Melt Flow Index-7.0 g/10 min, flow temperature: 190 °C, density: 0.959 g/cm^3^; Vicat softening temperature: 122 °C [30]), to which Heidelberg Cement’s SOLID 32.5 Portland cement [31] was added at 5 and 10% (by weight). The polymer material used for the tests was post-production waste obtained from a local manufacturer. In order to prepare the composites, the material in the form of granules was mechanically mixed with cement and introduced into the plasticizing system of the extruder, Rolbach SJ45, by means of which new material was produced using the parameters: cylinder heating temperature of zone I: 145 °C, zone II: 160 °C, zone III: 175 °C; the temperature of the extrusion head: 190 °C; auger rotation: 160 min^−1^. This material was then milled in a Shini SG-2417-CE slow speed mill. The obtained recyclate was used to produce test samples using a Kraus Maffei KM 65-160C1 injection molding machine. The injection moldings had the form according to the PN-EN ISO 527-1 standard [32].

Optimal properties of the injected samples (both HDPE and composites) were obtained under the following processing conditions: pressure limit in the plasticizing unit: 110 MPa, holding pressure: 60 MPa, holding time: 18 s, cooling time: 10 s, melt temperature: 190 °C, mould temperature: 80 °C.

Thermal DSC and degree of crystallinity tests of the samples were performed using a NETZSCH PC 200 scanning microcalorimeter, according to PN-EN ISO 11357-3:2018-06 [33]. DSC curves were recorded while heating the samples, in nitrogen atmosphere, at a rate of 10 °C/min in the temperature range 40–200 °C. he formulations for DSC studies were cut perpendicular to the flow direction from samples obtained by injection molding to minimize the skin-core effect. 

The Proteus software of the device was used to determine the degree of crystallinity; Polyma 214 NETZSCH software was used specifically. This program allowed studying the course of sample melting in the given temperature range and determining the area between the thermographic curve and baseline in the range of the occurrence of endothermic reflex. The mass of the samples was 11 mg. Samples were weighed with a SARTORIUS balance with an accuracy of 0.01 mg, with internal calibration and closed measuring space.

**Figure 1:**
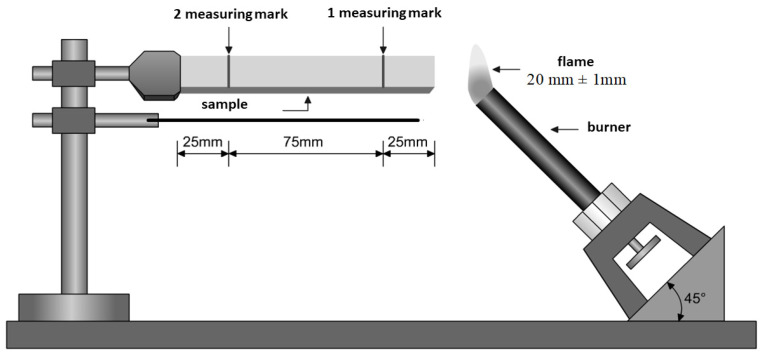
Schematic diagram of the apparatus for determining flammability by burner flame method UL 94HB [34].

Investigation of the material structure was conducted in transmitted light using an optical microscope, Nikon Eclipse E 200. The samples were microtomed slices with a thickness of 11 ÷ 14 μm that were cut from the core of the injection moulded parts. A rotary microtome Thermo Shandon Finesse Me+ was used for this purpose. Determination of the flammability of the polymeric material and comparison of the flammability of the composites were carried out on a test stand (Figure 1.) using UL 94 V, UL 94 HB methods. In the UL 94HB flammability test, the melt time is measured from the first to the second measurement point (the distance between the measurement points is 75 mm). If the molded part does not ignite within 30 s, the test is aborted. The time it takes for the material to burn within the measuring point is the result of the flammability of the test specimen. The specimens to be tested were made by injection molding. The specimen length was 127 mm, and the width was 12.7 mm. The thickness of the test specimen must not be more than 12.7 mm. Preparation of test specimens: the test material was conditioned for 48 h at 50% humidity and 23 °C.

**Figure 2:**
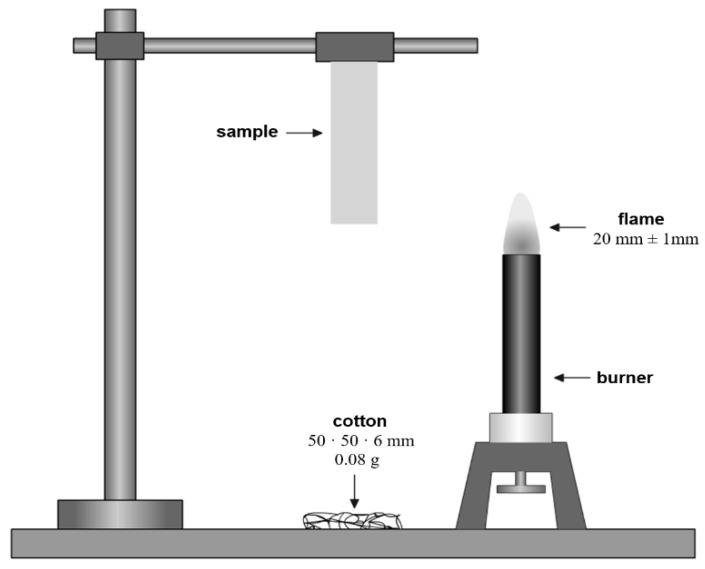
Schematic diagram of the apparatus for determining flammability with a burner by UL 94-V method [35].

In the UL-94-V method (Figure 2) for determining the flammability of the molded piece, a torch with a flame height of 20 ± 1 mm was used. The tested shape was subjected to the flame of the burner twice, each time for 10 s. The method consisted of moving the moving torch under the molding for 10 s. Then the flame was set aside for 7 s, after which it was moved under the molding again for another 10 s. The result of the measurement was the time it would take for the melting-lit shaper to ignite and burn the 0.08 g cotton just below it due to falling polymer droplets. If the tested shaped piece fails to ignite under the influence of the burner flame within a given time, the test is aborted. Table 1 shows the flammability classes defined by the UL 94-V method. The preconditioning of the fittings for the test consists of holding the fittings for 48 h at 50% humidity and 23 °C. 

The test specimens were made by injection molding. The length of the specimen was 127 mm, the width was 12.7 mm. The thickness of the test specimen must not be more than 12.7 mm.

A Hegewald & Peschke Meß- und Prüftechnik GmbH Inspekt 20 tensile testing machine was used to carry out the static tensile test, in which self-clamping jaws were set up for the static tensile test. The tests were carried out on standardized specimens according to EN ISO 527-2 [36], with dimensions of 150 × 10 × 4 mm. The test parameters were gauge length *L*_0_ = 110 mm, tensile speed *v* = 50 mm/s (according to PN-EN ISO 527-1 [36]. All samples were conditioned equally at room temperature. 

NETZSCH’s DMA 242 device was applied for dynamic mechanical properties tests, according to PN-EN ISO 6721-1:2019-07 [37], with a three-point free bending specimen holder in the form of a 50 × 10 × 4 mm beam. On the specimen placed in the holder, through the mandrel were introduced the actions of sinusoidally varying force with a frequency of 1 Hz and 10 Hz with constant amplitude of 120µm, while heating the specimen at a rate of 2 °C/min from a temperature of −150 °C to 100 °C. From the values of force and strain (read by measuring sensors), considering the dimensions of the specimen, the values of the storage modulus *E*′ and the loss modulus *E*′ and the loss tangent tanδ were calculated. The results are presented as a plot of the variation of the conservative modulus *E*’ and the loss tangent tanδ as a function of temperature.

## 3. Results and Discussion

**Figure 3:**
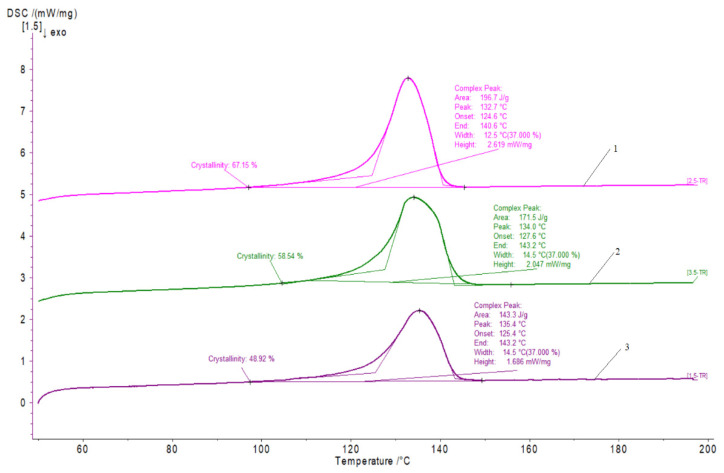
Example of DSC thermograms obtained in the first heating cycle (Netzsh Proteus program): (1) HDPE, (2) HDPE + 5% cement, (3) HDPE + 10% cement.

The results of DSC experiments are shown in Figure 3 and Table 2. Changes in the character of DSC curves and changes in the degree of crystallinity of the tested materials were found.

The DSC investigations prove the decrease in the crystallinity degree of the HDPE/cement composite after the cement addiction. However, as expected, the shape of the thermograms was insignificantly changed. Since HDPE is a semicrystalline polymer, a decrease in the crystalline phase after cement addiction was expected. The reduction in the value of the degree of crystallinity is probably due to the inhibition of the possibility of growth of spherulites after adding cement, which is confirmed by the microstructure tests.

For the composite samples, the temperature of the highest melting rate was not reduced, but the melting temperature range was changed significantly. With a higher cement content, a shift in the melting temperature range of the crystalline phase towards higher values was recorded.

In the case of composite samples, the amount of energy absorbed by the polymer is decreased. The melting enthalpy reached lowest values for the samples of HDPE with 10% of cement addiction. 

Other conditions for cooling the polymer by adding cement may cause too small a chain mobility. Therefore they can create areas with maximum ordering, leading to a reduction in the degree of crystallinity. However, slight undercooling favors the formation of more structured composite structures, as evidenced by microstructure studies.

**Figure 4:**
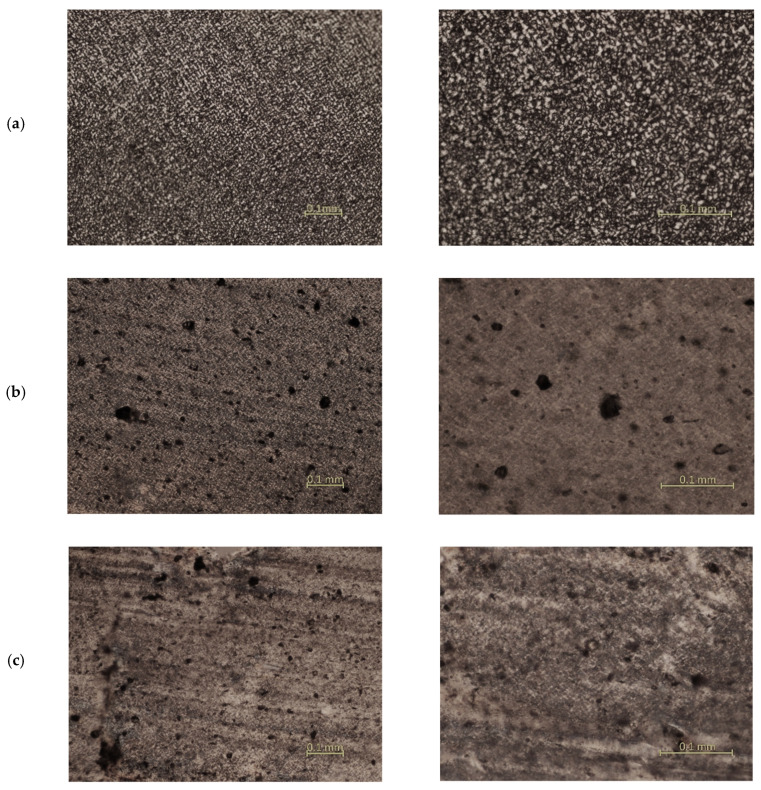
Structures from an optical microscope: (**a**) HDPE, (**b**) HDPE composite with 5% of cement, (**c**) HDPE composite with 10% of cement.

Figure 4 presents pictures of the microstructures for the polyethylene and composites.

Crystallization of the HDPE/cement composites proceeds through nucleation, the thermodynamically stable formation of embryos, and through a process of growth of the crystalline phase of polymer. The crystals grow faster in the pre-embryos created than evenly distributed throughout the amorphous phase. The emergence of any crystal growth process is initiated by the earlier formation of the embryo having a large surface area to its mass. The results of microscopic examination indicate that the nucleation is a heterogeneous composite produced by the presence of cement. The size of the crystal structures in the composite samples is smaller than samples of HDPE. In HDPE recyclate, spherulites have a size from 0.02 to 0.01 µm, while in samples with cement, they have a smaller size, from 0.01 to 0.006 µm. Adding cement blocks the development of crystalline structures. For the structure of a composite, characteristically, the size of the crystal structures decreases while their compaction increases in the case of higher cement content.

In this work, the flammability of polymeric material was determined, and the flammability of composites was compared using UL 94 V, UL 94 HB methods.

Thermal energy introduced to the polymer causes its heating and then melting. Further introduction of thermal energy leads to thermal decomposition processes: depolymerization, degradation, and destruction. High temperature evaporates the products of thermal decomposition (pyrolysis) and ignites the plastic. Sustaining the burning process depends on the amount of heat transferred from the flame to the plastic and the amount of flammable gases and oxygen in the environment. It follows that the combustion process will not be sustained if the decomposition of the plastic requires more heat than is supplied by the flame or if a solid, non-flammable residue covers the surface and isolates the flammable residue from the heat source.

The combustion of gases resulting from the thermal decomposition of a polymeric material is an exothermic reaction. The materials tested burn with a surface diffusion flame. The factor that determines the rate of flame combustion is the heat of combustion. The ignitability of the tested material is influenced by the content of hydrogen atoms in the polymer molecule. The lower the number of hydrogen atoms, the fewer flammable gases are produced during the thermal process. Due to its low molecular weight and high heat of combustion, hydrogen has the highest energy-to-mass ratio, and the explosion force of hydrogen is 2.5 times greater than conventional hydrocarbon fuels. Hydrogen is characterized by a low ignition energy and a high combustion rate. The mechanism of combustion of polymers depends on their structure. The combustion of thermoplastics is diffusion combustion (the speed of mixing the ingredients is much slower than the rate of chemical reactions), with the characteristics of homogeneous (gases) and heterogeneous (solid material and gases) combustion. The combustion process taking place in the zone separating the combustible gas from the air is controlled by the mutual diffusion of both components. The flammability of polymers is reduced by changing the energy balance of their combustion process, which is achieved by introducing additional components into the plastic, the so-called flame retardants. They are divided into two groups:

- additive, which are substances bound to the polymer because of physical type interactions—such as the cement used in the study;
- reactive substances, which join with polymer by chemical bonds.

Boron and molybdenum compounds, silicates, carbonates, and hydrated aluminum trioxide are used as additive flame retardants. On the other hand, reactive flame retardants are some organophosphorus, halogen and nitrogen-containing, organohalogen compounds, and antimony trioxide.

The filler used in the study acted as a flame retardant. In flammability tests, the HDPE was classified into flammability class V-2, while the composite with higher cement content approaches the V-0 flammability class, which proves the effect of the filler as a fire retardant of the composite produced based on recycled materials.

The flammability characteristics recorded in the UL 94 V flammability tests are shown in Table 3.

The following flammability characteristics were recorded in the UL 94 HB flammability tests:

(1) for HDPE:
  - burning time: 6 min 05 s (the sample burned with a high flame with a yellow glow and a blue core during the flaming test droplets dripped);
(2) for HDPE + 5% cement:
  - burning time 3 min 05 s (the sample burned with a low flame with a yellow glow and a blue core during the flaming test droplets dripped);
(3) for HDPE + 10% cement:
  - burning time 2 min 20 s (the sample burned with a low flame with a yellow glow and a blue core. As the sample burned, a large piece of material was left pointing downward, which burned before the droplet fell).

**Figure 5:**
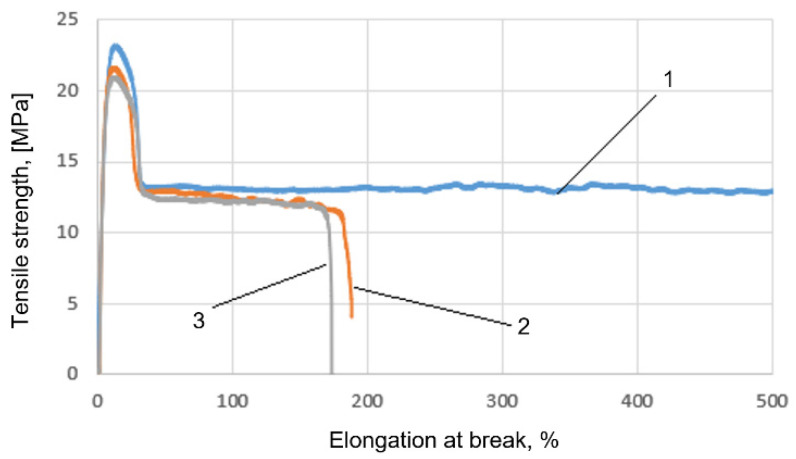
Example of diagram of the relation between tensile strength and elongation for: (1) HDPE, (2) HDPE + 5% cement (3) HDPE + 10% cement.

Figure 5 illustrates the results of the tensile strength tests. It was found that the addition of cement to the plastic caused a decrease in the elongation of the composites concerning HDPE. The above graphs (Figure 5) show that as the content of cement increases, the tensile strength of the molded parts decreases slightly. Parts from HDPE with a 5% cement content had a 6% lower tensile strength than unfilled parts, and successively, parts with 10% cement were 8.5% lower than the unfilled samples. The samples from HDPE without filler did not break, and those samples had much higher elongation at break than the samples with even fewer amounts of filler (higher than 500%). Higher quantities of cement additions reduced the extension of molded parts from polyethylene. Pieces with a 10% cement content exhibit the smallest elongation value, amounting to 173%.

**Figure 6:**
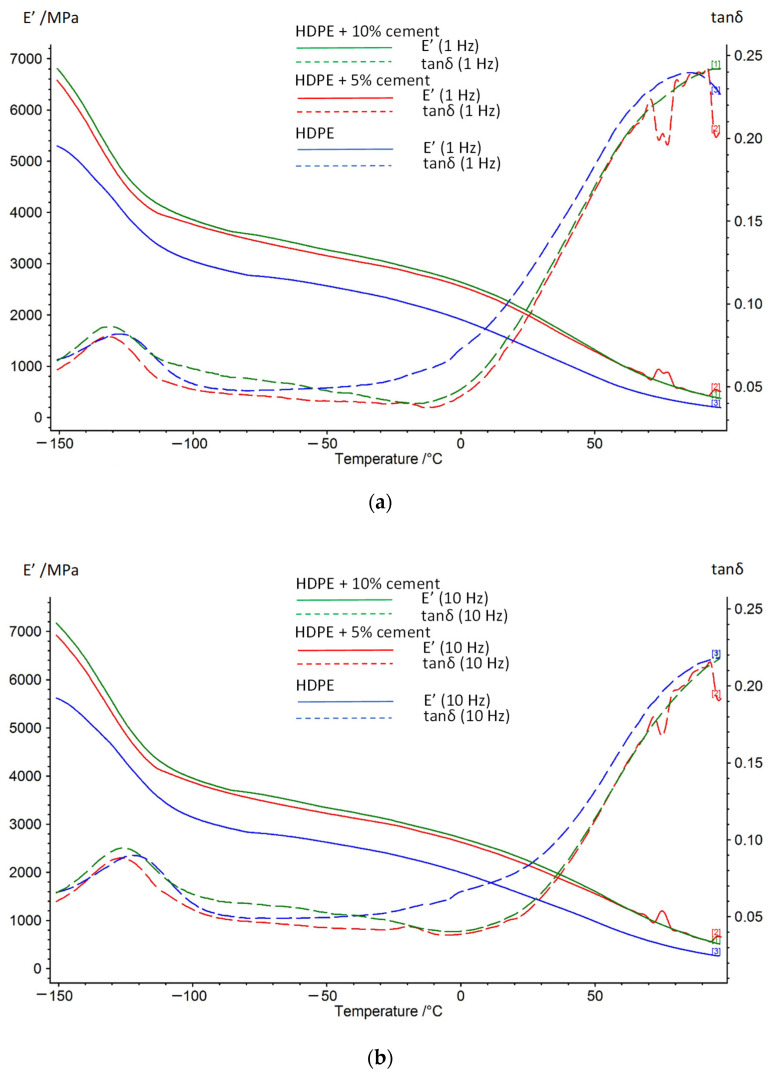
Example of DMTA plot of loss tangent tanδ and storage modulus E′ for (HDPE, HDPE + 5% cement, HDPE + 10% cement) for frequencies: (**a**) 1 Hz, (**b**) 10 Hz.

The results of thermomechanical properties are shown in Figure 6. The study shows that the addition of cement to polyethylene increases the e storage modulus values. The analysis of the recorded values of storage modulus and loss tangent tanδ shows the differences for the materials studied, but not significantly depending on the cement content in the composite. In the region of temperatures lower than the glass transition temperature, polyethylene is in a glassy state, and it is hard and brittle. In the glassy area, the thermal energy is insufficient to overcome the potential barrier rotational movement and displacement of the particle segments. The structure is in a state of thermodynamic imbalance. As the temperature increases, there is a decrease in the modulus value for HDPE. The material is in the glass transition region where the loss tangent reaches a maximum value at the glass transition temperature at a given strain frequency of 1 and 10 Hz. In the glass transition region, Brownian motion in the molecular chain is initiated. The thermal energy becomes comparable to the potential energy barrier for chain rotation. Near the glass transition temperature, the viscoelastic properties change very rapidly both with time and with changing temperature. By analyzing the changes in the values of the tangent of the loss tangent and the effect of cement addition on the material stiffness, differences in the values were found in the temperature range from −150 ℃ to −20 ℃, both at strain frequencies of 1 and 10 Hz and different filler additions. As the temperature increases, the polyethylene changes to a highly elastic state. In the temperature range from 20 ℃ to 100 ℃, the curves of HDPE and composites are similar to each other but differ significantly in values. In each case, the first reduction in E′ value corresponds to relaxation. The increase in the temperature leads to the second, substantial reduction in storage modulus that corresponds to the operation of relaxation of amorphous regions of HDPE. A flat profile zone can be observed for the curves within the range of temperature above vitrification temperature Tg, corresponding to the transition from the vitreous into the highly-plastic state. The increase in the temperature leads to a reduction in the value of module for composites. 

In the studied temperature range (from −150 to 100 °C), values E′ for the composites containing cement were considerably higher than those of E′ for HDPE without filler. The profile of changes in storage modulus versus temperature is similar for both frequencies studied (1 and 10 Hz). Storage modulus for the sample containing 10 wt% of filler at a temperature of 25 °C is 2070 MPa and is by 35% higher than E′ for HDPE, with a test frequency of 1 Hz. Therefore, the amorphous phase of the composite with 10 wt% of filler shows the strongest reinforcement with the particles of cement, which demonstrates a higher degree of dispersion of filler in the specimen compared other composites. It should be emphasized that the composites containing 5 and 10 wt% of filler are described by the similar and greater value of E′ over the whole temperature range compared to the non-filled polymer. Therefore, filler particles have a substantial reinforcing effect on the material for all compositions studied. The addition of cement causes a shift in a maximum of loss tangent tanδ towards higher temperatures. It reduces with the shift towards lower temperatures with the increase in cement content. These shifts in peaks result from the increasing stabilization of the composites resulting from the immobilization of the fragments of HDPE crystallites by the filler particles [38,39].

## 4. Conclusions

The addition of cement as a filler to polyethylene made it possible to obtain composites with good thermomechanical properties. The analyzed composites showed a decrease in the values of the degree of crystallinity, differences in the values of the melting point of the crystalline phase, a decrease in the elongation of the samples, and an increase in the values of the storage modulus of the samples compared to HDPE. The microstructure analysis of the composites confirmed the changes in the crystalline phase recorded in thermal studies by differential scanning calorimetry. The size of crystalline structures characteristically decreases for the composite, while their density increases with higher cement content. A significant effect of the filler as a flame retardant of the produced composite based on recycled materials was found in flammability tests.

The research results presented in the paper constitute an experimental cognitive element of the issue and require continuation and development in the aspect of testing composites with broader cement content.

## Figures and Tables

**Table 1:** Flammability classes as determined by UL 94-V [35].

Flammability Class	V-0	V-1	V-2
Burning time of specimen after burner application (s)	≤10	≤30	≤30
Total burning time (10 applications of flame) (s)	≤50	≤250	≤250
Burning time and glow after the second flame application (s)	≤30	≤60	≤60
Burning droplets appearance	yes	no	yes
Total combustion of specimen	no	no	no

**Table 2:** Results of DSC investigations obtained from calculations by Netzch program (averaged values from three measurements).

Material	Enthalpy [J/g]	Melt Temperature Range [°C]	Melt Temperature–Peak Max. [°C]	Crystallinity Degree [%]
HDPE	195.8	124.7 ÷ 140.6	132.7	66.82
HDPE + 5% of cement	170.1	125.4 ÷ 142.8	134.7	58.10
HDPE + 10% of cement	141.7	125.5 ÷ 143.3	135.3	48.36

**Table 3:** The UL 94 V flammability tests.

Material			
HDPE	burning time: 32 s	occurrence of burning drops: yes	complete combustion of the sample: yes
HDPE + 5% of cement	burning time 23 s	occurrence of burning drops: yes	complete burning of specimen: no
HDPE + 10% of cement	burning time less than 10 s	occurrence of burning drops: yes (sometimes)	complete burning of the sample no

## Data Availability

Data is contained within the article.
